# Severe Early Onset Obesity due to a Novel Missense Mutation in Exon 3 of the Leptin Gene in an Infant from Northwest India

**DOI:** 10.4274/jcrpe.5501

**Published:** 2018-07-31

**Authors:** Devi Dayal, Keerthivasan Seetharaman, Inusha Panigrahi, Balasubramaniyan Muthuvel, Ashish Agarwal

**Affiliations:** 1 Postgraduate Institute of Medical Education and Research, Department of Pediatrics, Division of Pediatric Endocrinology, Chandigarh, India; 2 Postgraduate Institute of Medical Education and Research, Department of Pediatrics, Division of Genetic-Metabolic, Chandigarh, India

**Keywords:** Congenital leptin deficiency, monogenic obesity, leptin gene, novel mutation, early onset obesity, India

## Abstract

Monogenic obesity, caused by mutations in one of the genes involved in the control of hunger and satiety, is a rare cause of early onset obesity (EOO). The most common of the single gene alterations affect the leptin gene (*LEP*), resulting in congenital leptin deficiency that manifests as intense hyperphagia, EOO and severe obesity associated with hormonal and metabolic alterations. Only eight mutations of *LEP* associated with congenital leptin deficiency have been described in humans to date. In this study, we report a novel, homozygous, missense mutation in exon 3 of the *LEP* gene (chr7:127894610;c.298G>A) resulting in the amino acid substitution of asparagine for aspartic acid at codon 100 (p.Asp100Asn) in a 10-month-old infant who presented to us with severe hyperphagia and EOO. She was subsequently found to have low serum leptin concentrations. Additionally, a homozygous missense variation of unknown significance in exon 11 of Bardet-Biedl syndrome-1 gene (chr11:66291279; G>A; Depth 168x) was detected. Significant abnormalities of lipid parameters were also present in our patient. Both parents were thin but there was a family history suggestive of EOO in a paternal uncle and a cousin. In conclusion, we report the second patient from India with a novel mutation of the *LEP* gene associated with severe obesity.

## What is already known on this topic?

Congenital leptin deficiency due to mutations of the leptin gene is a rare cause of early-onset obesity with less than 50 cases reported to date.

## What this study adds?

This article presents an Indian infant with severe early-onset obesity caused by a novel mutation in the leptin gene.

## Introduction

Severe early onset obesity (EOO) may be caused by alterations in genes that regulate appetite, body weight and energy homeostasis (1). The most common single-gene alterations that cause severe EOO include mutations in the leptin (*LEP*), leptin receptor (*LEPR*), preopiomelanocortin, prohormone convertase 1 or melanocortin 4 receptor genes, which together account for 3-5% of non-syndromic cases (1). These genes are involved in the control of hunger and satiety through the leptin-melanocortin signaling pathway in the hypothalamus. Of these genes, the most commonly affected is the *LEP* gene. Homozygous mutations in *LEP *cause the recessively inherited congenital leptin deficiency which manifests as severe EOO (1). Other characteristic manifestations include impaired satiety, intense hyperphagia, a normal birth weight and rapid weight gain during early infancy (1). These children also develop several hormonal and metabolic abnormalities associated with obesity in older children and adults (2). In addition, they may have reduced T-cell number and function, resulting in increased predisposition to infections and high rates of mortality during childhood (3). After the first report of a frameshift mutation in *LEP* in two severely obese cousins from a consanguineous United Kingdom family of Pakistani origin (4), several other patients with frameshift, missense or deletion mutations in *LEP* have been reported (5,6,7,8,9,10,11,12,13,14,15,16,17). These reports have emanated from several countries and especially from regions with high rates of consanguinity. Approximately 80% of about 50 patients described in the literature so far come from Central Pakistan (4,5,6,7,8). In this communication, we report a novel homozygous missense mutation in *LEP* associated with low serum leptin concentrations, hyperphagia and severe EOO in an infant from Northwest India.

## Case Report

A 10-month-old girl was referred to our endocrine unit for evaluation of excessive and rapid weight gain. She was born at full term by normal vaginal delivery and weighed 3.0 kg [-0.52 standard deviation score (SDS)] at birth. She is the second child of healthy, non-obese parents with third degree consanguinity. There was no history to suggest gestational diabetes, hypertension, hypothyroidism or excess weight gain by mother during pregnancy. Parents noticed increased appetite at about two months of age. She started demanding feeds at half to one hourly intervals. Subsequently, there was a rapid gain in her weight to 9.5 kg (+3.14 SDS) at four months and 15 kg (+8.17 SDS) at six months of age. There was no history of lethargy, dryness of skin, constipation, excessive hair growth, seizures, visual or sleep disturbances. There was a history of EOO in a paternal uncle and a male cousin.

**Figure 1:**
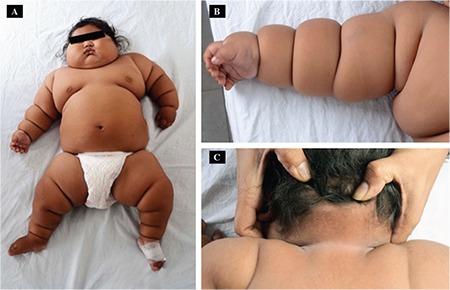
Clinical photographs of the patient showing A) generalized body fat distribution, B) deep skin folds and C) acanthosis nigricans

Physical examination revealed generalized body fat distribution, a rounded face and deep skinfolds (Figure 1A, 1B). There were no stigmata of a syndrome or underlying endocrinopathy, except acanthosis nigricans in axillae and neck folds (Figure 1C). The vital parameters were normal. Her weight was 19 kg (+7.38 SDS), length 71.0 cm (-0.24 SDS) and body mass index 37.7 kg/m^2^ (+10.94 SDS). Anthropometric calculations were done using the World Health Organizetion (WHO) Anthroplus software (version 1.0.4 WHO, Geneva, Switzerland). Ophthalmological evaluation did not show retinitis pigmentosa. Systemic examination was unremarkable.

Laboratory investigations revealed normal routine hematological and biochemical parameters, except for serum liver aminotransferases. The results of other laboratory evaluations are shown in Table 1. Abdominal ultrasound showed normal morphology of kidneys, a liver span of 12 cm (normal 6.3-9.6 cm) and features of hepatic steatosis. Magnetic resonance imaging of the brain, with fine cuts through the pituitary and hypothalamus, showed no abnormality. In view of intense hyperphagia followed by rapid weight gain, early age of onset, family history of EOO and low level of circulating leptin, a diagnosis of monogenic obesity due to *LEP* gene mutation was considered. Written informed consent was obtained from the parents for conducting all laboratory studies and for publishing clinical information and photographs.

**Figure 2:**
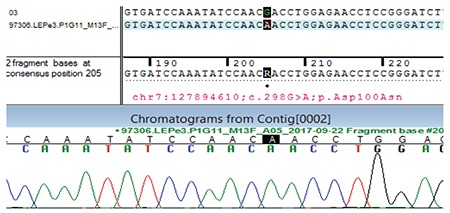
Sequence chromatogram showing homozygous missense mutation in exon 3 of the leptin gene (chr7:127894610;c.298G>A) resulting in the amino acid substitution of asparagine for aspartic acid at codon 100 (p.Asp100Asn)

For genetic studies, genomic DNA extracted from blood was used to perform targeted gene capture using a custom capture kit on Illumina HiSeq 2000 sequencing platform (Illumina, California, USA). Sequencing identified a homozygous missense mutation in exon 3 of the *LEP *gene (chr7:127894610;c.298G>A) resulting in the amino acid substitution of asparagine for aspartic acid at codon 100 (p.Asp100Asn). Validation of the identified mutation was done by Sanger sequencing to exclude false positivity (Figure 2). The Asp100Asn variant lies in the functional domain of the leptin protein and has not been reported in the 1000 Genomes database. It has a minor allele frequency of 0.0008% in the Exome Aggregation Consortium (ExAC) database. The *in silico* predictions of the effect of the mutation are “probably pathogenic” by Polyphen-2 (HumDiv) and “pathogenic” by Sorting Intolerant From Tolerant, Log ratio test and MutationTaster2. The reference codon is conserved across species.

Sequencing also revealed a homozygous missense variation in exon 11 of Bardet-Biedl syndrome-1 (*BBS1*) gene (chr11:66291279; G>A; Depth 168x) resulting in amino acid substitution of isoleucine for valine at codon 346 (p.Val346Ile). This variant has a minor allele frequency of 0.16% and 0.1% in the 1000 Genomes and ExAC databases respectively. The *in silico* prediction of the effect of the mutation is “pathogenic” by only MutationTaster2. The reference codon is conserved across mammals. This *BBS1 *mutation is classified as a variant of uncertain significance based on the above evidence. Sanger sequencing of exon 3 of the *LEP *gene and exon 11 of *BBS1 *gene of the unaffected parents identified the same variations as in the index patient but with heterozygous inheritance.

## Discussion

The majority of children with EOO have simple obesity (18). However, about 5% of all children with EOO may have monogenic obesity caused by mutations in one of the several genes involved in the regulation of appetite and body weight (1). Even rarer are the syndromic forms of EOO such as BBS, Prader-Willi syndrome and Beckwith-Wiedemann syndrome caused by genetic, epigenetic and genomic alterations (1). The most common and treatable form of monogenic obesity is due to mutations in the *LEP* gene manifesting as hyperphagia and rapid weight gain starting from early infancy (4). The clinical manifestations in the index patient were similar to the previously reported patients (4,5,6,7,8). Additionally, our patient exhibited severe abnormalities of lipid parameters, usually found in patients with congenital leptin deficiency during late childhood or even adulthood, at 10 months of age (8). However, other common obesity-associated complications such as abnormalities of glucose homeostasis and blood pressure were not detected in our patient (2,8). The mild elevation of serum aminotransferases was possibly related to hepatic steatosis commonly seen in patients with obesity and dyslipidemia (19).

The mutation (chr7:127894610;c.298G>A) in our patient that led to amino acid substitution of asparagine for aspartic acid at codon 100, has not been described previously. However, a different missense variation (Asp100Tyr) affecting the same codon has been reported (13). Interestingly, the affected patient had high circulating levels of mutant leptin (functional studies showed that leptin was biologically inactive) (13), unlike the characteristically absent or nearly absent circulating leptin in *LEP* gene mutations (5,6,7,8).We presume that the low serum leptin concentrations secondary to the mutated *LEP* gene resulted in severe hyperphagia and severe EOO in our patient. The serum concentrations of leptin were even lower than the recent local normative data for children (mean serum levels 1.4±0.5, range 1.04-3.71 ng/mL) (20).

Leptin is an important afferent, peripheral, humoral signal to the appetite-regulating network in the hypothalamus and affects food intake and energy expenditure. It is an important predictor of weight gain even during early infancy (21). Therefore, low levels of leptin or its biological inactivity resulting from mutations in *LEP *may disturb metabolic balance, leading to severe obesity and related metabolic disorders. Leptin replacement normalises these hormonal and metabolic alterations, suggesting that leptin deficiency or inactivity is the predominant determinant of obesity associated disorders in these patients (3,9,13).

The finding of *BBS1* gene mutation in our patient is intriguing. BBS is a known cause of a syndromic form of EOO and BBS proteins are required for *LEPR* signalling (22). However, leptin resistance rather than leptin deficiency is the characteristic finding in obese patients with BBS (23). The obesity usually manifests by 2-3 years of age, unlike that found in patients with *LEP* gene mutation which manifests in early infancy (22,23). Furthermore, our patient did not show the usual BBS stigmata, such as retinitis pigmentosa, kidney dysfunction, polydactyly, behavioural problems and hypogonadism (22). Low levels of circulating leptin and compromised leptin signalling may account for the extreme obesity seen in this case.

A significant majority of the previously reported patients belong to consanguineous families of the Arain tribe, who live in the Central Punjab, Pakistan (5,6,7). Incidentally, our patient hails from a geographical area in Indian Punjab approximately 30 miles away from this location, where most patients so far described live (5,6,7). Although families of the Arain community have a scattered presence across Northern India, including Punjab, their consanguinity rates are lower. Our patient does not belong to the Arain community, although third degree consanguinity was present. The first reported patient from India also came from North India (16). The geographic location points to either the operation of natural selection (carrier advantage) or random genetic drift (chance founder effects) for *LEP *gene in this population.

The limitations of our study include the lack of functional studies to understand the mechanism of disease manifestations in the patient. Also, we could not screen other affected family members for the mutation detected in our patient.

In summary, we report an infant with congenital leptin deficiency due to a novel mutation of the *LEP *gene, manifesting as severe EOO and dyslipidemia. This is only the second case from India with *LEP* gene mutation in the published literature. In patients with EOO, identifying those with *LEP* gene mutations is important, as recombinant human leptin therapy offers substantial clinical benefits in these patients.

## Figures and Tables

**Table 1:**
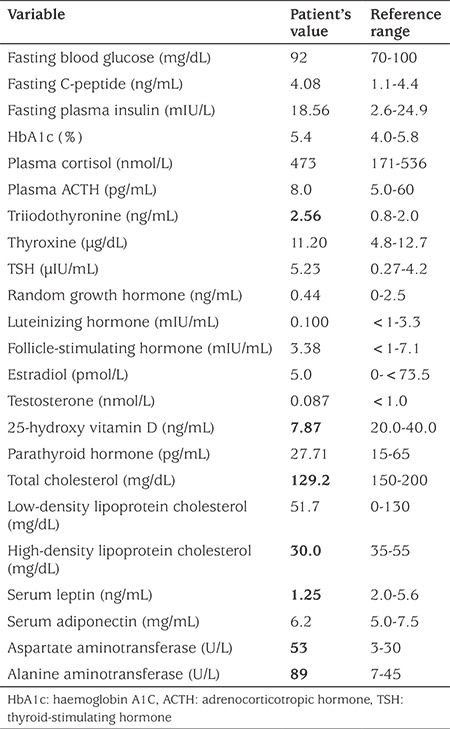
Hormonal, metabolic and other laboratory results of the patient at presentation
